# Machine Learning‐Based Model for Predicting Recurrence‐Free Survival After Interventional Therapy in Malnourished Hepatocellular Carcinoma Patients

**DOI:** 10.1002/cam4.71157

**Published:** 2025-09-14

**Authors:** Ningning Lu, Chunwang Yuan, Bin Sun, Xiongwei Cui, Wenfeng Gao, Yonghong Zhang

**Affiliations:** ^1^ Interventional Therapy Center for Oncology Beijing You'an Hospital, Capital Medical University Beijing China

**Keywords:** CONUT score, machine learning, malnourished hepatocellular carcinoma patients, nomogram, recurrence free survival

## Abstract

**Objective:**

This study intends to utilize machine learning approaches to screen out the crucial factors affecting the recurrence of hepatocellular carcinoma (HCC) patients with preoperative malnutrition after interventional therapy, and based on the identified factors, develop a nomogram for predicting the patients' 1‐, 3‐, and 5‐year recurrence‐free survival (RFS).

**Methods:**

This study encompassed the clinical data of 512 malnourished (CONUT score ≥ 2) HCC patients who received the combination treatment of transarterial chemoembolization (TACE) and radiofrequency ablation (RFA) at Beijing You'an Hospital between January 2014 and January 2020. These patients were then randomly partitioned into training and validation cohorts at a 7:3 ratio. To investigate the factors influencing the post‐treatment recurrence of malnourished HCC patients, methods such as random survival forest (RSF), eXtreme gradient boosting (XGBoost), and multivariate Cox regression analysis were employed. A nomogram was constructed based on the identified crucial factors to predict RFS in HCC patients. Subsequently, its performance was evaluated through Kaplan–Meier (KM) curves, receiver operating characteristic curve (ROC), calibration curve, and decision curve analysis (DCA).

**Results:**

This study determined that GGT, APTT, age, and ALT are independent risk factors influencing recurrence in malnourished HCC patients. Based on the four risk factors, a nomogram for predicting RFS was effectively developed. The KM curve analysis showed that the nomogram could significantly distinguish between patient groups with varying recurrence risks. Furthermore, the nomogram's discriminative ability, accuracy, and decision‐making efficacy were validated through the above‐mentioned evaluation indicators, collectively suggesting its robust predictive performance.

**Conclusions:**

We developed a nomogram that can predict the 1‐, 3‐, and 5‐year RFS of malnourished HCC patients after undergoing the combination treatment; the constructed nomogram exhibited favorable predictive capabilities.

## 1 Introduction

Hepatocellular carcinoma (HCC) is one of the most common malignant tumors worldwide, with its incidence and mortality rates continuing to rise in recent years. HCC is the sixth most common cancer globally and the third leading cause of cancer‐related deaths [1]. Interventional therapies, such as transarterial chemoembolization (TACE) combined with radiofrequency ablation (RFA) therapy, can effectively reduce tumor volume and prolong patient survival by blocking tumor blood supply and directly ablating tumor tissue, and have become one of the primary treatment options for HCC patients [2, 3, 4]. However, although these treatments can effectively control tumor growth in the short term, the long‐term recurrence rate of HCC remains high, significantly impacting patients' quality of life and overall survival [5]. Studies have shown that recurrence occurs in up to 70% of patients within 5 years after the initial treatment, and this recurrence can be a result of residual tumor cells, microvascular invasion, or the underlying liver dysfunction [6, 7, 8]. Therefore, understanding the risk factors for recurrence is critical for optimizing patient management, guiding post‐treatment surveillance, and tailoring follow‐up strategies to improve long‐term outcomes.

The Controlling Nutritional Status (CONUT) score is a nutritional assessment tool based on serum albumin, cholesterol, and lymphocyte count, which has been widely used to evaluate patients' nutritional status [9]. Patients having CONUT scores in the range of 0–1 exhibit a normal nutritional status. Conversely, those with CONUT scores from 2 to 4 are at risk of light malnutrition. Patients with scores between 5 and 8 are at risk of moderate malnutrition, while those with scores of 9–12 are at risk of severe malnutrition [10]. The CONUT score is not only related to patients' nutritional status but also closely associated with the prognosis of various malignancies [11, 12, 13, 14]. Recent studies have demonstrated that the CONUT score can be a valuable prognostic marker in HCC, with higher scores correlating with poorer survival rates, greater risk of recurrence, and more severe liver dysfunction [15, 16, 17]. However, there is currently a lack of studies specifically focusing on developing recurrence prediction models for HCC patients with high CONUT scores. Most existing research has either broadly examined HCC recurrence predictors or explored the general prognostic value of the CONUT score without tailoring predictive models to this high‐risk subgroup. This gap in the literature highlights the need for conducting in‐depth research on the risk factors for recurrence in HCC patients with high CONUT scores and constructing effective predictive models.

In this study, we focus on developing a machine learning‐based model to predict recurrence in malnourished HCC patients (CONUT score ≥ 2) following interventional therapy. By leveraging the strengths of XGBoost and random survival forest (RSF), we can extract underlying patterns from multidimensional data, offering a new solution for assessing recurrence risk. This study represents a critical step toward addressing the unmet need for accurate recurrence prediction in this vulnerable population, paving the way for more targeted and effective clinical interventions.

## 2 Materials and Methods

### 2.1 Patient Selection

This research received ethical approval (ethical approval number: Beijing You'an Hospital Research Ethics No. 2022073) from the ethics committee of Beijing You'an Hospital, affiliated with Capital Medical University. All methodologies complied with the hospital's ethical guidelines for human studies and aligned with the principles of the Helsinki Declaration. Given the retrospective design of the study, the use of de‐identified patient information, and the negligible risk involved, the ethics committee granted an exemption from obtaining informed consent.

In this retrospective research, the data of 690 patients who were diagnosed with HCC and received a combination treatment of TACE and RFA at Beijing You'an Hospital, between January 2014 and January 2020, were first examined. Then, 178 patients were excluded from the study based on the exclusion criteria. Ultimately, a total of 512 malnourished patients were included in this study following the specified inclusion criteria. The combined treatment was carried out by physicians with a minimum of 5 years of specialized experience in the field. TACE was first employed to target and treat the lesions by directly delivering chemotherapy drugs along with embolic agents into the blood vessels supplying the tumor. Within a timeframe of one to 2 weeks following the TACE procedure, RFA was performed to destroy any remaining cancer cells and ensure comprehensive treatment of the affected area.

**FIGURE 1:**
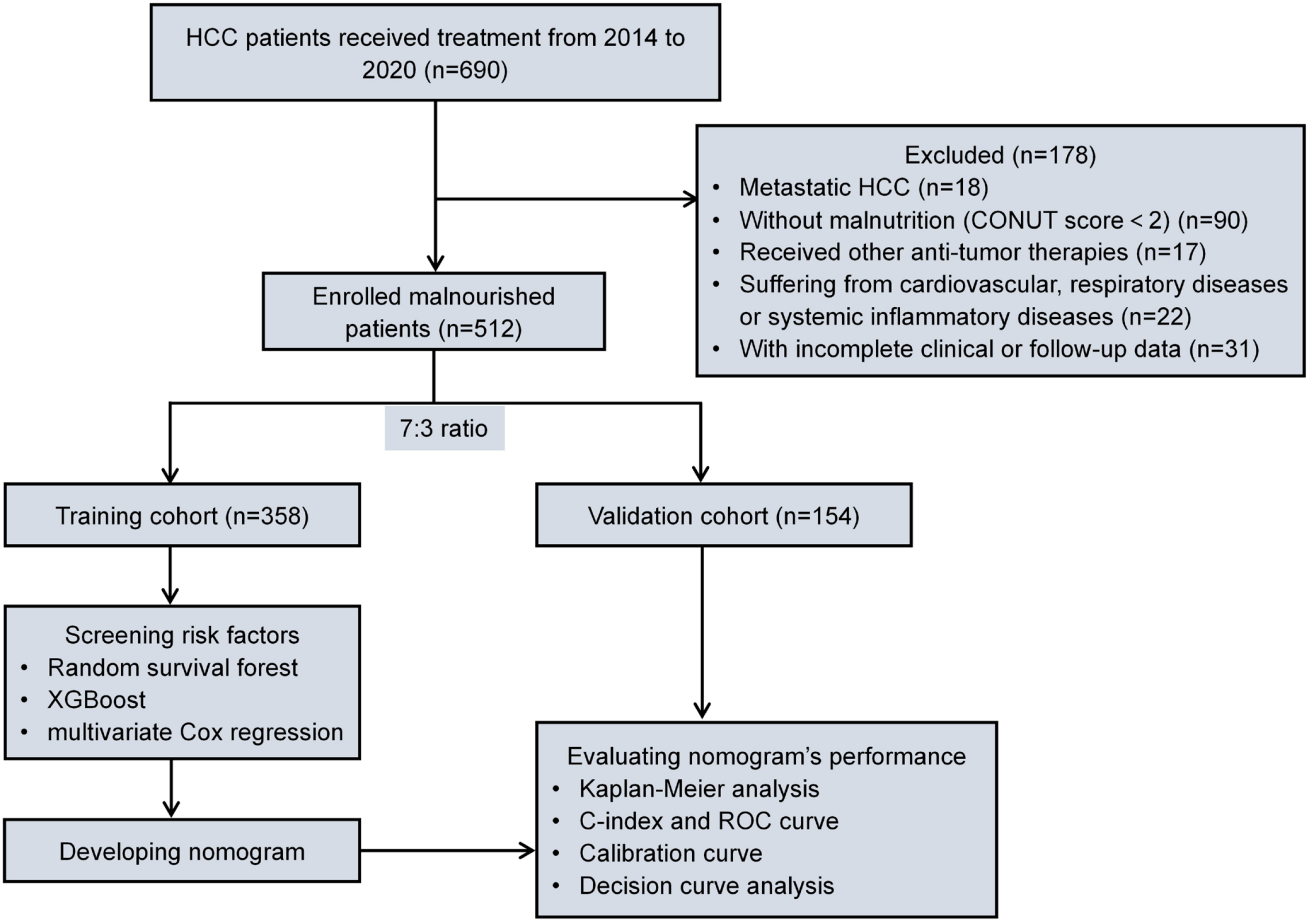
Patient selection and study design. CONUT, controlling nutritional status; HCC, hepatocellular carcinoma; ROC, Receiver Operating Characteristic Curve; XGBoost, eXtreme gradient boosting.

The following are the inclusion and exclusion criteria of this study. Inclusion criteria: (1) Patients with a diagnosis of primary HCC; (2) Those who received the combined treatment of TACE and RFA; (3) Patients with malnutrition (CONUT score ≥ 2); (4) Without pre‐operative surgical resection, immunotherapy, and so on; (5) Having complete clinical and follow‐up data. Exclusion criteria: (1) Cases that are metastatic HCC; (2) CONUT score < 2; (3) Patients who received other anti‐tumor therapies prior to the combined treatment; (4) Those suffering from cardiovascular or respiratory diseases or inflammation; (5) Cases with incomplete clinical or follow‐up data. The process of patient enrollment and study design is illustrated in Figure 1.

### 2.2 Data Collection

The collected patient data can be categorized into the following groups: demographic information (age, gender), medical history (hypertension, diabetes, cirrhosis, family history of liver cancer), lifestyle factors (smoking history, antiviral history), liver function assessment (Child–Pugh, ALT, AST, AST/ALT, TBIL, DBIL, GGT, ALP, prealbumin, bile acid), blood routine indicators (WBC, neutrophil, monocyte, RBC, hemoglobin, platelet), coagulation profile (PT, APTT, fibrinogen, TT), tumor characteristics (tumor number, tumor size, BCLC, AFP), metabolic indicators (glucose, uric acid, potassium, sodium, chloride), and protein metabolism (total protein, globulin). These data comprehensively reflect the patient's health status, disease progression, and treatment‐related metrics. By analyzing this information, clinicians can evaluate liver function, tumor burden, metabolic status, and coagulation function, providing a basis for conducting subsequent analysis to screen out the most statistically significant factors for recurrence. Factors demonstrating significant associations in the machine learning analysis will be further evaluated in the multivariate Cox regression to control for potential confounders. Based on the results, we will establish a prognostic nomogram to predict patients' RFS.

### 2.3 Follow‐Up and Study Endpoint

After undergoing the combined treatment, patients are advised to adhere to a structured follow‐up schedule. The initial follow‐up is typically scheduled approximately 1 month post‐treatment to assess the efficacy of the therapy. Subsequently, follow‐up visits are recommended quarterly during the first year post‐treatment, transitioning to biannual visits thereafter, until patient death or loss to follow‐up. During these follow‐up appointments, doctors will monitor the patient's progress and evaluate any potential side effects or complications. Diagnostic tests, such as blood work, imaging studies, or other relevant assessments, may be conducted to ensure the treatment's continued effectiveness and to detect any signs of recurrence or new health concerns. RFS is the endpoint of this study. It is the time from the start of combination treatment to the first recurrence or the last follow‐up. Tumor recurrence is defined as the detection of typical tumor manifestations via follow‐up imaging tests like contrast‐enhanced CT or MRI after treatment or as being confirmed by pathological examination. The last follow‐up date of this study is December 31, 2023.

### 2.4 Statistical Analysis

In this study, we employed a range of statistical methods to analyze and interpret the data. Continuous variables were summarized using means and standard deviations to capture both central tendency and variability, while categorical variables were described using frequencies and percentages to highlight the distribution of categories across the sample. The dataset was randomly split into training and validation cohorts in a 7:3 ratio to ensure robust model development and evaluation. To compare groups, we applied the *t*‐test for continuous variables and the chi‐squared test for categorical variables, with statistical significance set at *p* < 0.05. To identify critical factors associated with RFS in HCC patients following treatment, we adopted advanced machine learning methods (random survival forest and XGBoost) and multivariate Cox regression. Using the key predictors identified through these methods, we developed a prognostic nomogram to predict RFS. Patients were stratified into low‐risk and high‐risk groups based on the optimal cutoff value derived from the nomogram scores. For survival analysis, we utilized the Kaplan–Meier method to estimate the cumulative probability of RFS over time, and the log‐rank test was used to compare survival curves between different risk groups. To evaluate the overall performance of the model, we calculated several statistical metrics, including the receiver operating characteristic (ROC) curves to assess discrimination ability, calibration curves to evaluate the agreement between predicted and observed outcomes, and decision curve analysis (DCA) to determine the clinical utility of the model. These comprehensive evaluations ensured that the model was not only statistically sound but also clinically relevant. All analyses were conducted using R version 4.3.2.

## 3 Results

### 3.1 Baseline Characteristics of the Enrolled Patients

This study included a training cohort of 358 patients and a validation cohort of 154 patients. The two cohorts were well‐balanced in terms of various baseline characteristics, as indicated by the non‐significant *p* values (Table 1). In terms of demographic features, the proportion of male patients was 71.2% in the training cohort and 72.1% in the validation cohort (*p* = 0.93). Regarding comorbidities, the prevalence of hypertension, diabetes, and a history of antiviral use was similar between the two groups. For example, the rate of hypertension was 23.2% in the training cohort and 24.0% in the validation cohort (*p* = 0.926). Liver‐related characteristics such as cirrhosis status, Child–Pugh class, and BCLC stage also showed no significant differences. The proportion of patients with cirrhosis was 89.1% in the training cohort and 89.6% in the validation cohort (*p* = 0.989). Laboratory parameters like blood cell counts, liver function tests, and coagulation indicators were comparable. For instance, the mean values of ALT in the training and validation cohorts were 31.93 ± 20.36 U/L and 32.68 ± 21.31 U/L respectively (*p* = 0.707). APTT, an important coagulation index, had a mean value of 33.79 ± 5.01 s in the training cohort and 32.97 ± 3.96 s in the validation cohort (*p* = 0.073). These results suggest that the two cohorts are comparable at baseline, which provides a reliable foundation for further analysis and validates the generalizability of the study findings.

**TABLE 1:** Baseline characteristics of training and validation cohorts.

Characteristic	Training cohort (*N* = 358)	Validation cohort (*N* = 154)	*p*
Gender (male/female)	255 (71.2%)/103 (28.8%)	111 (72.1%)/43 (27.9%)	0.93
Hypertension (no/yes)	275 (76.8%)/83 (23.2%)	117 (76.0%)/37 (24.0%)	0.926
Diabetes (no/yes)	298 (83.2%)/60 (16.8%)	122 (79.2%)/32 (20.8%)	0.337
Antiviral history (no/yes)	150 (41.9%)/208 (58.1%)	65 (42.2%)/89 (57.8%)	1
Smoking history (no/yes)	249 (69.6%)/109 (30.4%)	104 (67.5%)/50 (32.5%)	0.727
Family history of liver cancer (no/yes)	188 (52.5%)/170 (47.5%)	83 (53.9%)/71 (46.1%)	0.849
Cirrhosis (no/yes)	39 (10.9%)/319 (89.1%)	16 (10.4%)/138 (89.6%)	0.989
Child–Pugh class (A/B)	259 (72.3%)/99 (27.7%)	115 (74.7%)/39 (25.3%)	0.663
BCLC stage (0/A/B)	114 (31.8%)/186 (52.0%)/58 (16.2%)	41 (26.6%)/90 (58.4%)/23 (14.9%)	0.383
Tumor number (single/multiple)	256 (71.5%)/102 (28.5%)	101 (65.6%)/53 (34.4%)	0.218
Tumor size (≤ 3 cm/> 3 cm)	247 (69.0%)/111 (31.0%)	100 (64.9%)/54 (35.1%)	0.425
Age	57.05 ± 9.41	55.75 ± 9.43	0.152
WBC (10^9^/L)	4.94 ± 2.04	5.10 ± 2.04	0.399
Neutrophil (10^9^/L)	3.27 ± 1.74	3.47 ± 1.91	0.235
Monocyte (10^9^/L)	0.39 ± 0.22	0.40 ± 0.22	0.871
RBC (10^9^/L)	4.14 ± 0.62	4.07 ± 0.60	0.218
Hemoglobin (g/L)	128.80 ± 18.53	127.77 ± 19.65	0.574
Platelet (10^9^/L)	114.73 ± 57.15	123.61 ± 65.22	0.123
ALT (U/L)	31.93 ± 20.36	32.68 ± 21.31	0.707
AST (U/L)	32.38 ± 15.94	33.24 ± 15.85	0.574
ALT/AST	1.20 ± 0.58	2.03 ± 10.15	0.121
TBIL (μmol/L)	19.76 ± 10.00	19.35 ± 9.01	0.664
DBIL (μmol/L)	6.69 ± 4.45	6.81 ± 4.44	0.78
Total protein (g/L)	64.74 ± 6.00	64.22 ± 7.67	0.405
Globulin (g/L)	28.08 ± 5.33	27.97 ± 5.18	0.828
GGT (U/L)	61.21 ± 52.78	67.45 ± 60.08	0.24
ALP (U/L)	86.15 ± 31.06	89.45 ± 34.73	0.287
Prealbumin (g/L)	129.88 ± 55.84	131.85 ± 55.35	0.714
Bile acid (μmol/L)	21.60 ± 29.24	24.51 ± 34.57	0.329
Uric acid (μmol/L)	271.33 ± 85.85	282.60 ± 93.91	0.186
Glucose (mmol/L)	5.89 ± 1.99	7.08 ± 13.63	0.107
Potassium (mmol/L)	3.92 ± 0.39	3.99 ± 0.39	0.074
Sodium (mmol/L)	139.67 ± 2.80	138.86 ± 11.29	0.205
Chloride (mmol/L)	103.82 ± 3.38	104.32 ± 4.21	0.157
PT (s)	12.82 ± 1.64	12.70 ± 1.38	0.454
APTT (s)	33.79 ± 5.01	32.97 ± 3.96	0.073
Fibrinogen (g/L)	2.84 ± 0.96	2.78 ± 0.94	0.494
TT (s)	15.75 ± 2.20	15.92 ± 2.16	0.406
AFP (ng/mL) (≥ 400/< 400)	44 (12.3%)/314 (87.7%)	20 (13.0%)/134 (87.0%)	0.397

Abbreviations: AFP, alpha‐fetoprotein; ALP, alkaline phosphatase; ALT, alanine aminotransferase; APTT, activated partial thromboplastin time; AST, aspartate aminotransferase; BCLC, Barcelona Clinic Liver Cancer; DBIL, direct bilirubin; GGT, gamma glutamyl transpeptidase; PT, prothrombin time; RBC, red blood cell; TBIL, total bilirubin; TT, thrombin time; WBC, white blood cell.

### 3.2 Determining the Factors That Influence RFS Through RSF, XGBoost, and Multivariate Cox Regression

**FIGURE 2:**
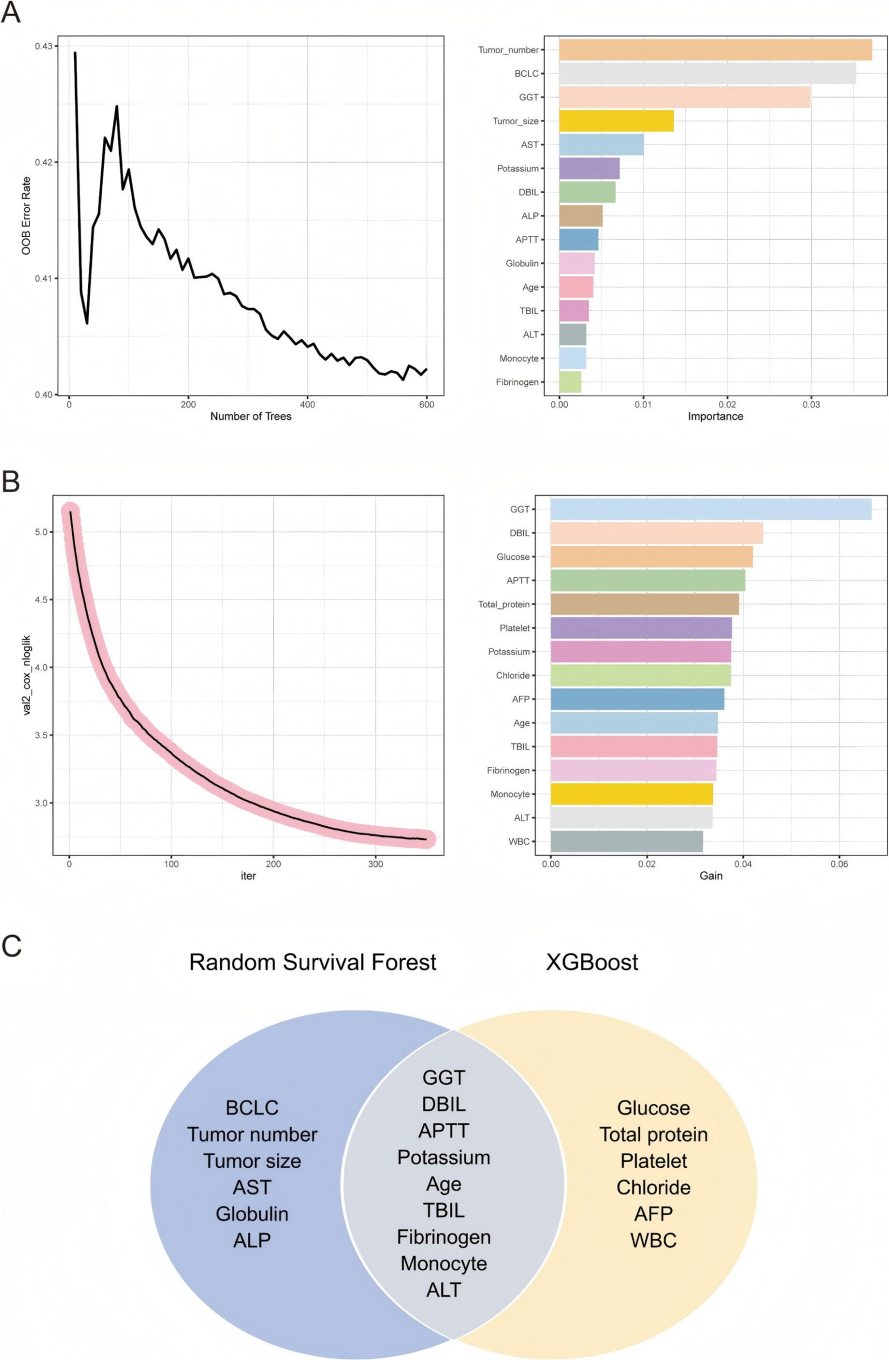
Determining the factors that influence RFS. (A) Determination of significant predictors for RFS through random survival forest. (B) Determination of significant predictors for RFS through eXtreme Gradient Boosting. (C) Visualization of overlapping variables between random survival forest and eXtreme Gradient Boosting. AFP, alpha‐fetoprotein; ALP, alkaline phosphatase; ALT, alanine aminotransferase; APTT, activated partial thromboplastin time; AST, aspartate aminotransferase; BCLC, Barcelona Clinic Liver Cancer; DBIL, direct bilirubin; GGT, gamma glutamyl transpeptidase; TBIL, total bilirubin; WBC, white blood cell.

To comprehensively identify the factors influencing RFS in patients with HCC following treatment, we employed a multi‐step analytical approach integrating machine learning techniques and classical statistical methods. We first employed the RSF algorithm. RSF is a non‐parametric ensemble learning method that can handle complex relationships between variables and survival outcomes. By using RSF, we were able to rank variables according to their importance in predicting RFS. This allowed us to screen out variables that had little to no impact on recurrence. As shown in Figure 2A, the top 15 important variables obtained from RSF analysis include tumor number, BCLC, GGT, tumor size, AST, potassium, DBIL, ALP, APTT, globulin, age, TBIL, ALT, monocyte, and fibrinogen.

Subsequently, the XGBoost algorithm was utilized. XGBoost is a powerful gradient‐boosting framework that can also effectively handle high‐dimensional data and capture non‐linear relationships. It provided another set of variable importance rankings for predicting RFS. Figure 2B visually presents the top 15 key variables selected by the XGBoost algorithm, including GGT, DBIL, glucose, APTT, total protein, platelet, potassium, chloride, AFP, age, TBIL, fibrinogen, monocyte, ALT, and WBC.

After obtaining the variable rankings from both RSF and XGBoost, we took the intersection of the important variables identified by these two methods. This intersection step was crucial as it helped to filter out variables that might be over‐emphasized by only one method and focused on the variables that were consistently important across different machine‐learning approaches. Figure 2C shows the intersection results, including GGT, DBIL, APTT, potassium, age, TBIL, fibrinogen, monocyte, and ALT.

**FIGURE 3:**
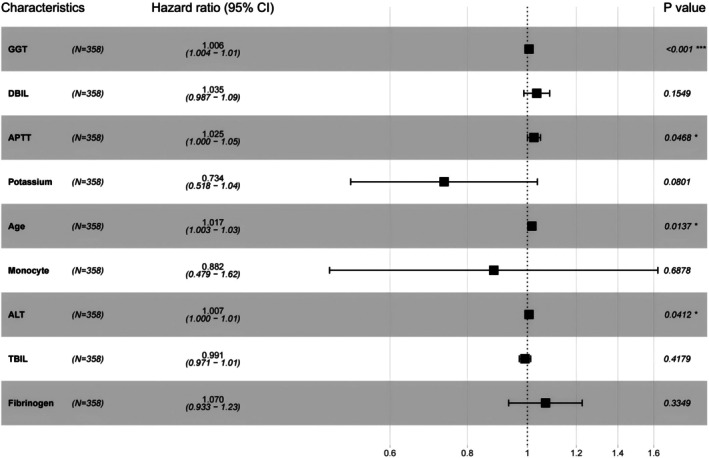
Results of the multivariate Cox regression analysis. ALT, alanine aminotransferase; APTT, activated partial thromboplastin time; DBIL, direct bilirubin; GGT, gamma glutamyl transpeptidase; TBIL, total bilirubin. *p < 0.05; ***p < 0.001.

Finally, the variables in the intersection were entered into a multivariate Cox regression analysis. Cox regression, a semi‐parametric model, is widely used in survival analysis to assess the relationship between covariates and the hazard of an event (in this case, recurrence). By adjusting for potential confounders, multivariate Cox regression provided hazard ratios (HRs) and 95% confidence intervals (CIs) for each predictor, offering a clear understanding of their impact on RFS. Figure 3 presents that through multivariate Cox regression, GGT, APTT, Age, and ALT were identified as independent risk factors affecting RFS. This comprehensive approach enabled us to identify the key factors that truly influence RFS in HCC patients after treatment.

### 3.3 Developing Nomogram for RFS Prediction Based on the Factors

**FIGURE 4:**
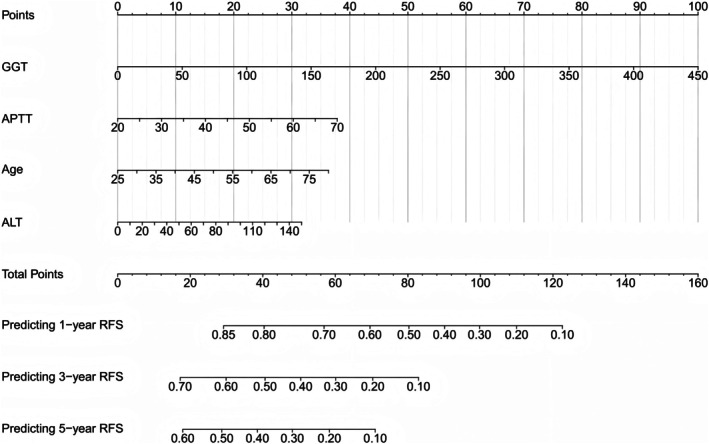
Nomogram for predicting 1‐, 3‐, and 5‐year RFS. ALT, alanine aminotransferase; APTT, activated partial thromboplastin time; GGT, gamma glutamyl transpeptidase; RFS, recurrence‐free survival.

Based on the identified factors including GGT, APTT, Age, and ALT, we developed a nomogram for predicting 1‐, 3‐, and 5‐year RFS (Figure 4). Leveraging the results from the multivariate Cox regression analysis, the relative contributions of each factor to RFS were determined. These were then converted into a point‐based scoring system. For each patient, the values of GGT, APTT, Age, and ALT are translated into corresponding points. Summing these points allows for an estimation of the 1‐, 3‐, and 5‐year RFS probabilities, presenting a user‐friendly tool for clinicians to make prognosis predictions.

### 3.4 Comparing the Kaplan–Meier Curves of Low and High‐Risk Groups Segmented by the Nomogram Scores

**FIGURE 5:**
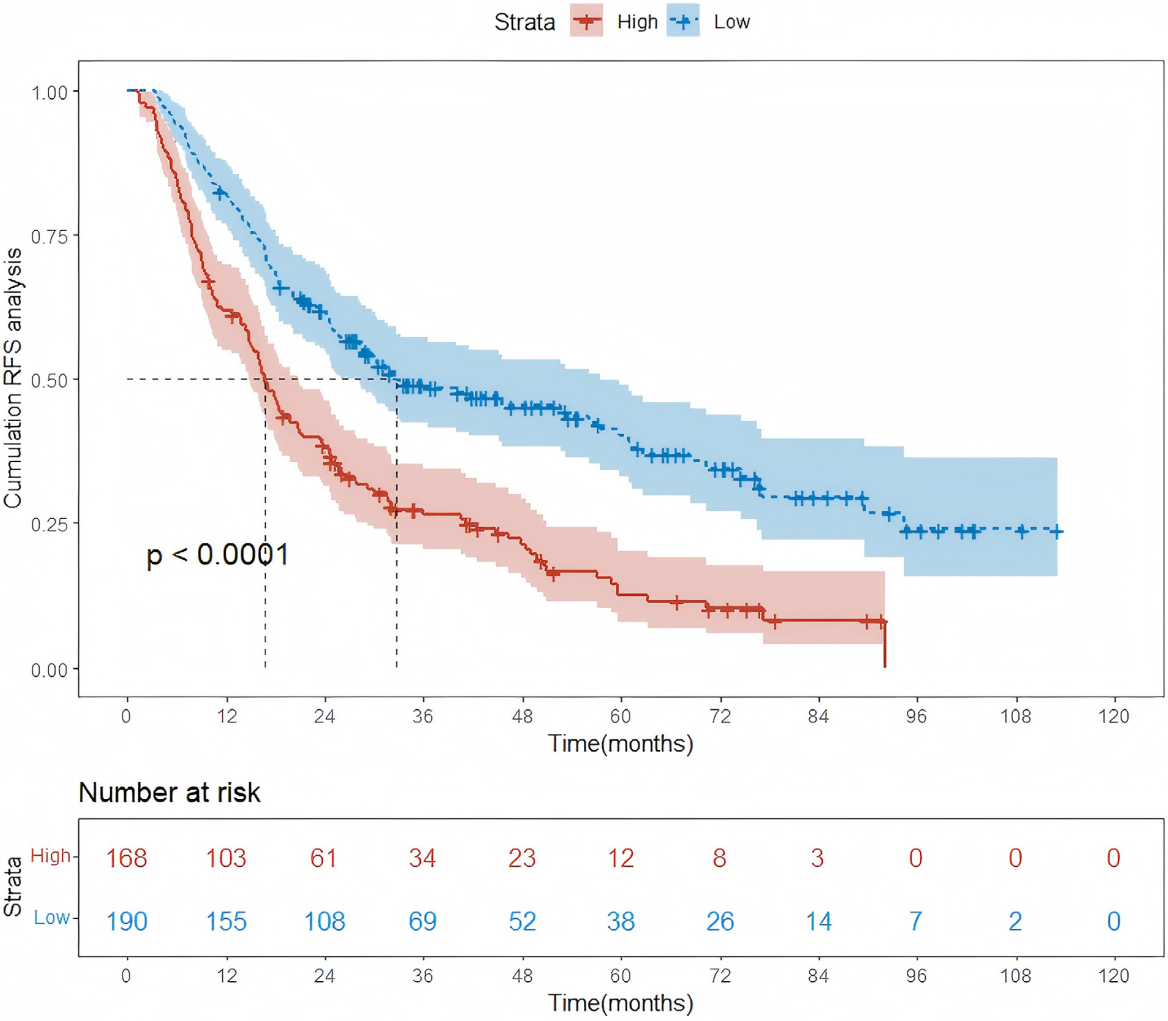
Comparison of Kaplan–Meier curves in the training cohort. RFS, recurrence free survival.

After establishing the nomogram for predicting RFS in HCC patients post‐treatment, we determined the optimal cutoff values for the nomogram scores in the training and validation cohorts. We employed the surv_cutpoint function in R to compute the optimal cutoff value. This function, part of the survminer package, is designed to identify the best threshold for dichotomizing continuous variables in survival analysis. Using these cutoff values, patients were segregated into low‐risk and high‐risk groups. It can be seen in both the training (Figure 5) and validation cohorts (Figure S1) that the curve for the low‐risk group typically showed a more favorable RFS, with a slower decline in the proportion of patients remaining recurrence free over time. In contrast, the high‐risk group's curve dropped more steeply, indicating a significantly higher recurrence rate. These visual differences in the Kaplan–Meier curves clearly demonstrated the nomogram's ability to stratify patients according to their risk of recurrence, validating its predictive utility in clinical practice.

### 3.5 Evaluating the Performance of the Nomogram

After the development of the nomogram for predicting recurrence of HCC patients, assessing its predictive performance in the training and validation cohorts is of great importance. This evaluation encompasses three key aspects: discrimination, calibration, and clinical utility.

**FIGURE 6:**
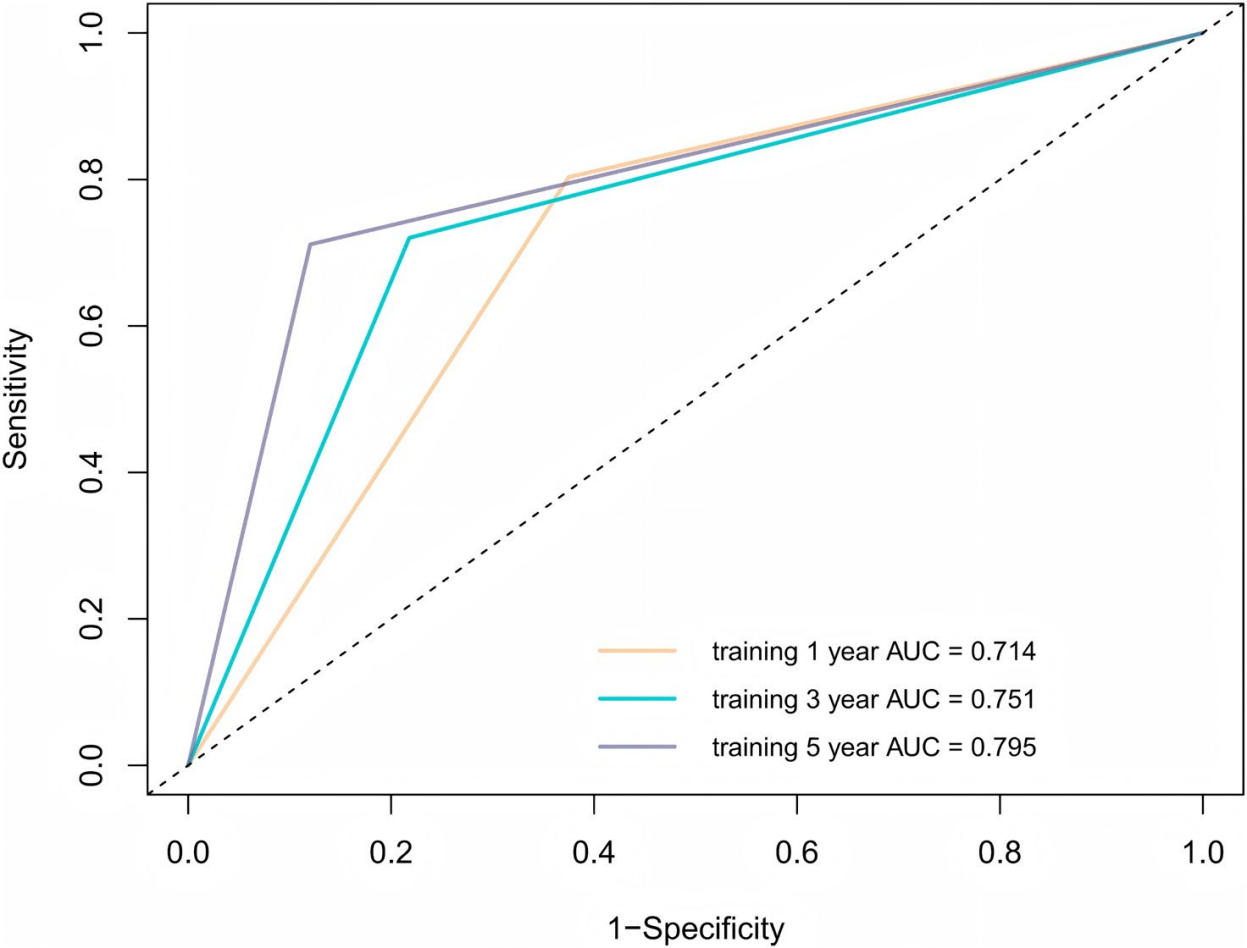
Receiver operating characteristic (ROC) curve of the nomogram in the training cohort. AUC, area under the curve.

Discrimination measures the nomogram's ability to distinguish between patients who will experience recurrence and those who will not. We evaluated this using the Area Under the ROC Curve (AUC). In the training cohort, for 1‐year RFS, the nomogram achieved an AUC of 0.714; as the time horizon extended to 3‐ and 5‐year RFS, the AUC values were 0.751 and 0.795 respectively (Figure 6). In the validation cohort, the AUCs for 1‐, 3‐, and 5‐year RFS were 0.690, 0.706, and 0.745 (Figure S2). These values, all well above 0.5, clearly indicated that the nomogram had a strong discriminatory power. A higher AUC implied that the nomogram can accurately distinguish patients at different risk levels of recurrence, providing valuable information for clinical decision‐making.

**FIGURE 7:**
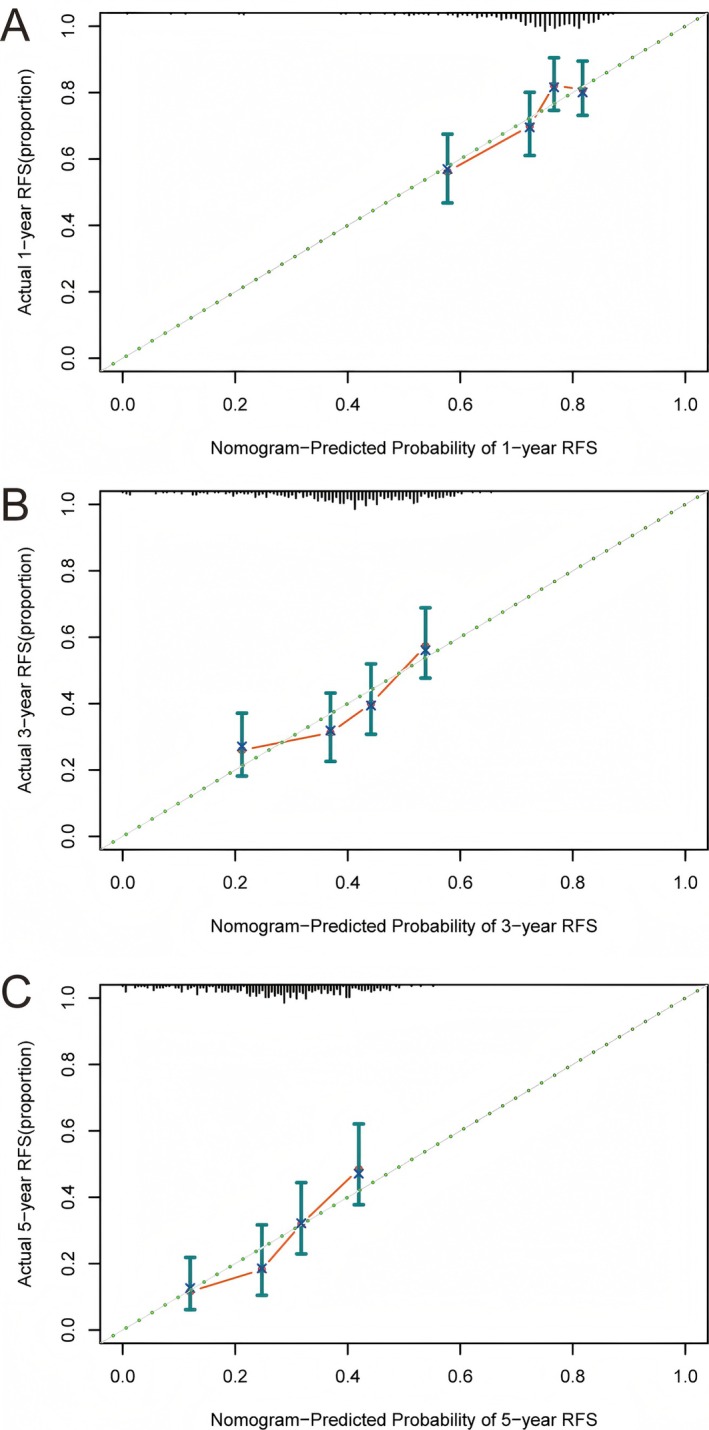
Calibration curves of the nomogram in the training cohort. (A) Calibration curve of 1‐year RFS prediction. (B) Calibration curve of 3‐year RFS prediction. (C) Calibration curve of 5‐year RFS prediction. RFS, recurrence‐free survival.

Calibration determines how well the predicted probabilities by the nomogram match the actual observed events. We constructed calibration curves for 1‐, 3‐, and 5‐year RFS. A well‐calibrated nomogram would have a calibration curve that closely follows the 45° line, where the predicted probability equals the actual probability. In our analysis, the calibration curves for the nomogram in the training cohort showed a relatively close proximity to the 45° line (Figure 7). This meant that the nomogram was reliable in estimating the probabilities of recurrence at different time intervals, giving clinicians confidence in the predicted values. In the validation cohort, the calibration curves also maintained a satisfactory close fit to the 45° line (Figure S3). This consistency in calibration across different datasets validated the nomogram's ability to provide reliable probability estimates.

**FIGURE 8:**
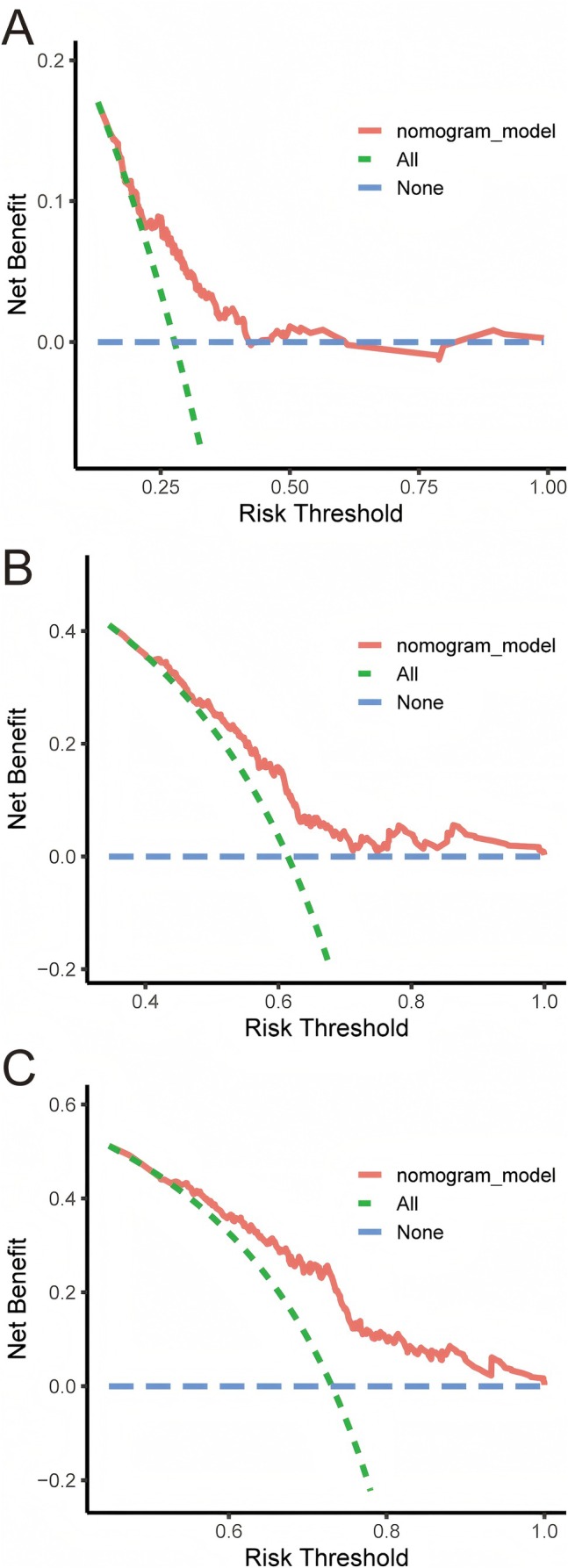
Decision curve analysis (DCA) of the nomogram in the training cohort. (A) DCA curve of 1‐year RFS prediction. (B) DCA curve of 3‐year RFS prediction. (C) DCA curve of 5‐year RFS prediction. RFS, recurrence‐free survival.

The clinical utility of the nomogram was evaluated using the DCA curves. DCA assesses the net benefit of a prediction model across a range of threshold probabilities. The DCA curves for the nomogram in the training cohort demonstrated that, within a reasonable range of threshold probabilities, the nomogram provided a higher net benefit compared to both the assumption that all patients will experience recurrence and the assumption that no patients will experience recurrence (Figure 8). The same was true for the validation cohort (Figure S4). This indicated that the nomogram could be effectively used in clinical decision‐making, helping clinicians to make more informed choices regarding patient management.

## 4 Discussion

In this study, we endeavored to develop and validate a machine learning‐based nomogram for predicting recurrence after intervention therapy in malnourished HCC patients.

There were four indicators that were eventually screened and incorporated into our prognostic model, namely, GGT, APTT, age, and ALT. Many previous studies have demonstrated the significant value of these indicators in assessing the prognosis of HCC patients. GGT, an enzyme reflecting liver metabolism and function, has been proven to be closely related to the occurrence and development of liver cancer. A higher GGT level often indicates a more severe degree of liver cell damage and an increased risk of tumor recurrence [18]. APTT reflects the body's coagulation function. Existing research has pointed out that elevation in APTT can serve as a potential indicator for predicting poor prognosis in cancer patients [19]. Age is also a non‐negligible factor in the prognostic assessment of HCC patients. As age increases, the immune function and liver reserve capacity of patients decline, which increases the likelihood of tumor recurrence. ALT is another important indicator of liver function. Abnormal ALT levels indicate liver inflammation activity and are associated with the risk of HCC recurrence [20].

Many previous studies have predominantly focused on the CONUT score as a prognostic factor for predicting the overall prognosis of patients. In moderate/severe aortic stenosis, a high CONUT score is linked to all‐cause death; worse nutrition indicates a higher risk [21]. The CONUT score is also a promising tool for predicting post‐transplant outcomes in multiple myeloma patients, with higher scores linked to delayed neutrophil engraftment and increased risk of oral mucositis [22]. A meta‐analysis of 15 studies revealed that higher CONUT scores are strongly associated with increased risks of major adverse cardiovascular events, mortality, cardiac death, myocardial reinfarction, and atrioventricular block, highlighting the significance of the CONUT score in predicting outcomes for acute myocardial infarction patients [23]. A retrospective study analyzed the clinical data of 2553 patients who underwent radical surgery for colorectal cancer. It discovered that the higher the preoperative CONUT score, the greater the risk of postoperative pulmonary complications, all‐cause mortality, and the worse the surgical outcomes, such as ICU admission, longer hospital stay, and 1‐year mortality [24]. Chen's research showed that for patients with intermediate‐stage HCC who received TACE, the CONUT score was an important prognostic factor for overall survival and progression‐free survival. These investigations have firmly established that a high CONUT score is associated with a poor prognosis [25]. However, despite the wealth of research on the prognostic value of the CONUT score, there has been a conspicuous absence of research on recurrence‐prediction models specifically tailored to this vulnerable group (malnourished/CONUT score ≥ 2), as this group is more likely to have a worse prognosis. Most of the existing research in the field of recurrence prediction in HCC has been rather general [26, 27, 28, 29, 30], often not stratifying patients based on nutritional status as measured by the CONUT score. This lack of targeted research is a significant gap, considering that HCC patients with CONUT scores ≥ 2, who are nutritionally compromised, may have different biological behaviors, responses to interventional therapies, and recurrence patterns compared to the general population.

Our study filled this void by developing a machine learning‐based model for predicting recurrence after interventional therapy in this specific high CONUT score HCC patient population. The results of our analysis hold implications for both clinical practice and the broader field of HCC research. Numerous studies in predicting patients recurrence have predominantly relied on traditional statistical methods. For example, many early works only used univariate and multivariate Cox regression to identify risk factors for recurrence [31, 32, 33]. These studies typically focused on a limited set of clinical variables. While these models have provided valuable insights, they often struggle to capture complex relationships among variables. In contrast, our machine learning approach, which includes algorithms like RSF and XGBoost, can handle non‐linear associations between multiple factors, leading to more accurate predictions.

Nevertheless, our study also has its limitations. Our model's generalizability may be constrained by the relatively small sample size of malnourished patients in the dataset. Additionally, as this is a single‐center retrospective study, the broader applicability of the model still warrants exploration. In the future, we plan to collaborate with other hospitals to conduct multi‐center studies. In clinical practice, patient data is continuously updated. Our model does not currently integrate real‐time data, such as new laboratory test results during follow‐up. Real‐time data integration is an important step in the future for translating the model into routine clinical practice. Incorporating emerging biomarkers, such as circulating tumor DNA and epigenetic markers, could enhance the model's predictive accuracy. Long‐term prospective studies are also needed to evaluate the real‐world impact of using our model on patient survival and treatment‐related morbidity.

## 5 Conclusions

We developed a nomogram that can predict the 1‐, 3‐, and 5‐year RFS of malnourished HCC patients after undergoing the combination treatment; the constructed nomogram exhibited favorable predictive capabilities.

## Author Contributions

**Ningning Lu:** conceptualization, methodology, writing – original draft, writing – review and editing, data curation. **Chunwang Yuan:** data curation, investigation, validation. **Bin Sun:** data curation, investigation, validation. **Xiongwei Cui:** data curation, investigation, validation. **Wenfeng Gao:** data curation, investigation, validation. **Yonghong Zhang:** conceptualization, methodology, project administration, writing – review and editing, supervision.

## Ethics Statement

The present study was approved by the ethics committee of Beijing You'an Hospital, affiliated with Capital Medical University.

## Consent

The necessity for securing written informed consent was waived, as the risk of the study was minimal and the identities of the patients involved were maintained in strict anonymity.

## Conflicts of Interest

The authors declare no conflicts of interest.

## Supporting information


**Figure S1:** Comparison of Kaplan–Meier curves in the validation cohort. RFS, recurrence free survival.

**Figure S2:** Receiver operating characteristic (ROC) curve of the nomogram in the validation cohort. AUC, area under the curve.

**Figure S3:** Calibration curves of the nomogram in the validation cohort. (A) Calibration curve of 1‐year RFS prediction. (B) Calibration curve of 3‐year RFS prediction. (C) Calibration curve of 5‐year RFS prediction. RFS, recurrence‐free survival.

**Figure S4:** Decision curve analysis (DCA) of the nomogram in the validation cohort. (A) DCA curve of 1‐year RFS prediction. (B) DCA curve of 3‐year RFS prediction. (C) DCA curve of 5‐year RFS prediction. RFS, recurrence‐free survival.

## Data Availability

The data that support the findings of this study are available from the corresponding author upon reasonable request.
