# Supplementary figures and images for: Machine Learning‐Based Model for Predicting Recurrence‐Free Survival After Interventional Therapy in Malnourished Hepatocellular Carcinoma Patients
Source: Cancer Med. 2025 Sep 14;14(18):e71157. doi: 10.1002/cam4.71157 (PMC12433893; doi:10.1002/cam4.71157)

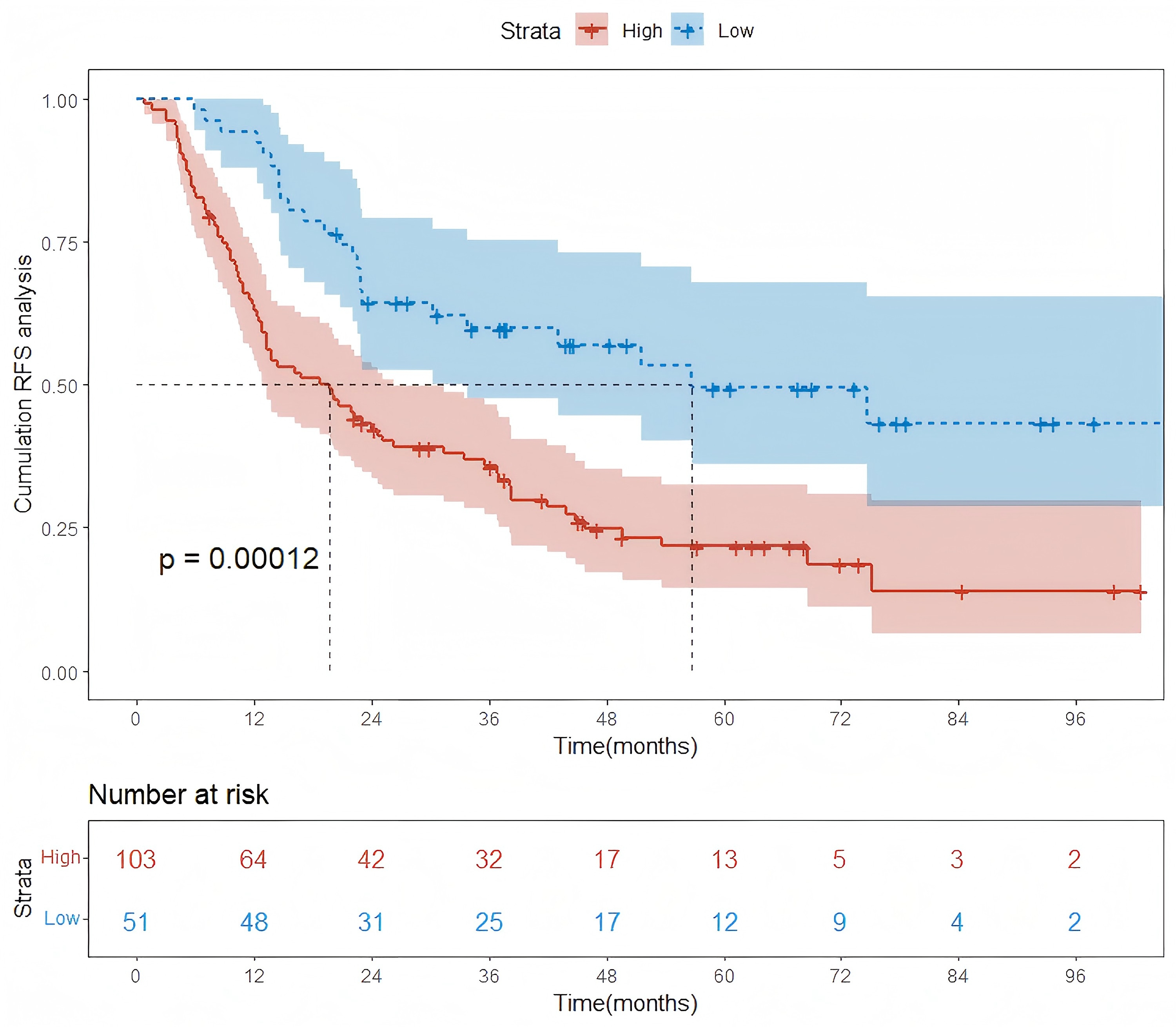

Supplement: Supplementary file 1 — Figure S1: Comparison of Kaplan–Meier curves in the validation cohort. RFS, recurrence free survival. [file CAM4-14-e71157-s004.jpg]

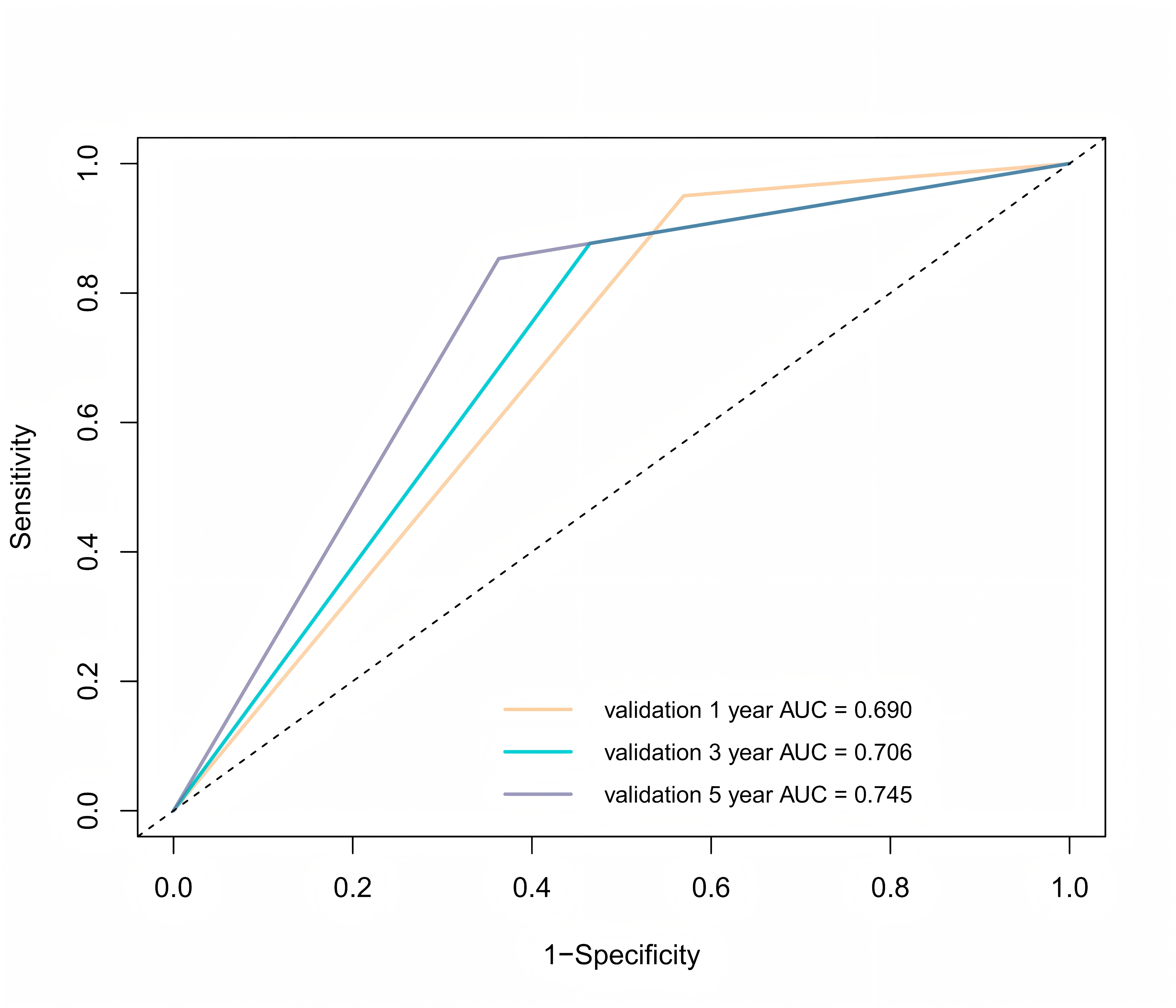

Supplement: Supplementary file 2 — Figure S2: Receiver operating characteristic (ROC) curve of the nomogram in the validation cohort. AUC, area under the curve. [file CAM4-14-e71157-s003.jpg]

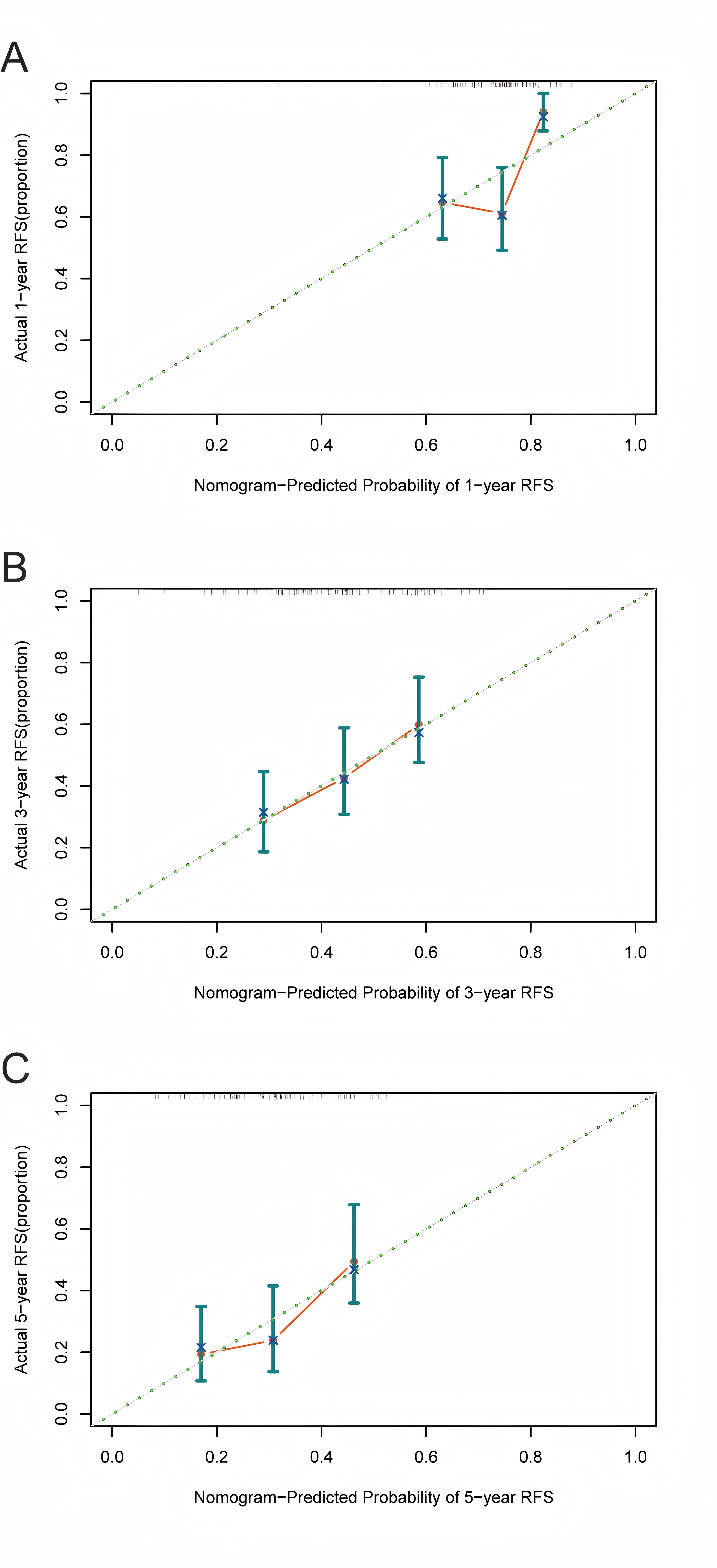

Supplement: Supplementary file 3 — Figure S3: Calibration curves of the nomogram in the validation cohort. (A) Calibration curve of 1‐year RFS prediction. (B) Calibration curve of 3‐year RFS prediction. (C) Calibration curve of 5‐year RFS prediction. RFS, recurrence‐free survival. [file CAM4-14-e71157-s001.jpg]

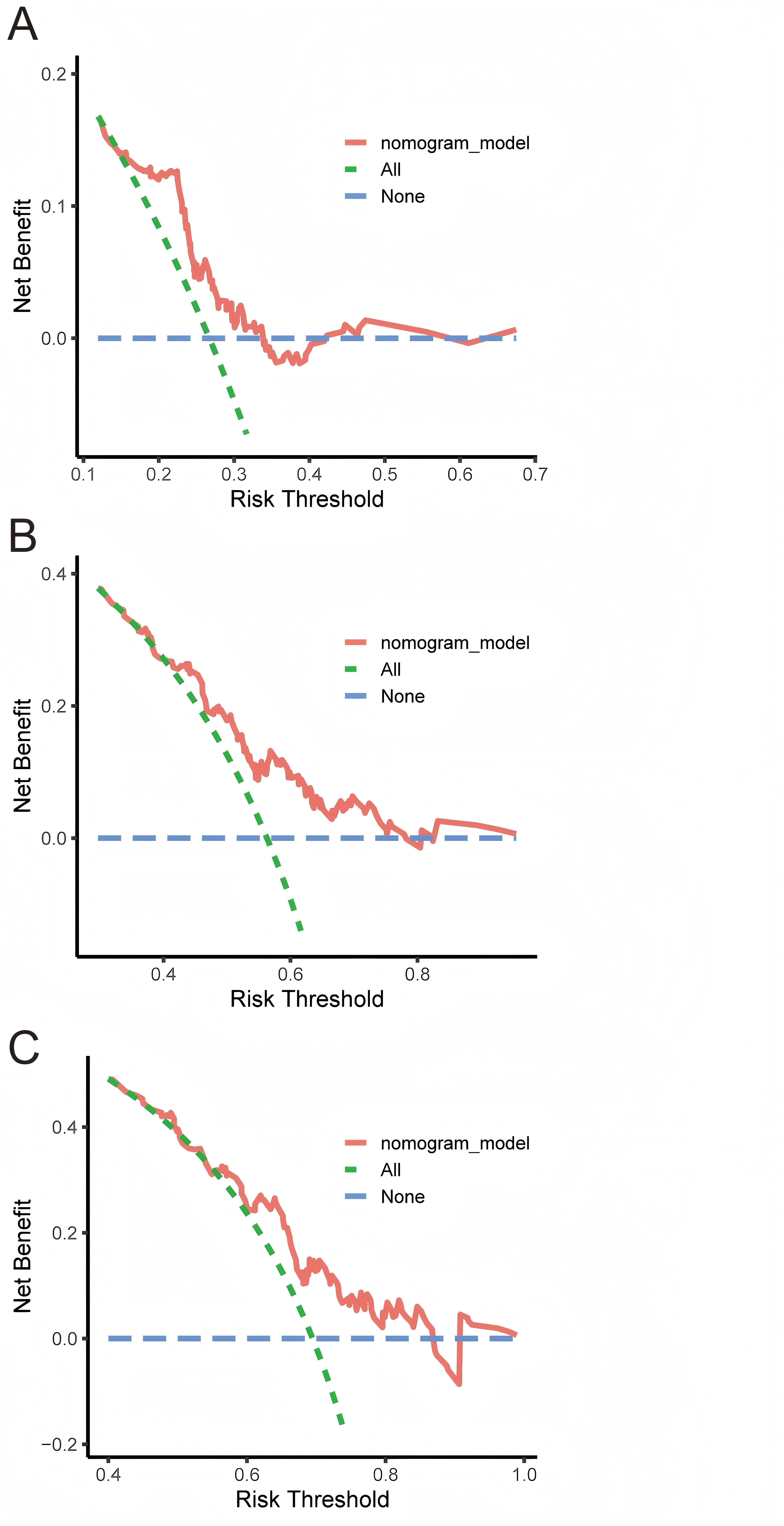

Supplement: Supplementary file 4 — Figure S4: Decision curve analysis (DCA) of the nomogram in the validation cohort. (A) DCA curve of 1‐year RFS prediction. (B) DCA curve of 3‐year RFS prediction. (C) DCA curve of 5‐year RFS prediction. RFS, recurrence‐free survival. [file CAM4-14-e71157-s002.jpg]
